# NOD1 Cooperates with TLR2 to Enhance T Cell Receptor-Mediated Activation in CD8 T Cells

**DOI:** 10.1371/journal.pone.0042170

**Published:** 2012-07-27

**Authors:** Blandine C. Mercier, Erwan Ventre, Marie-Laure Fogeron, Anne-Laure Debaud, Martine Tomkowiak, Jacqueline Marvel, Nathalie Bonnefoy

**Affiliations:** 1 Université de Lyon, Lyon, France; 2 Institut National de la Santé et de la Recherche Médicale, U851, Lyon, France; 3 Université Lyon 1, Lyon, France; 4 Hospices Civils de Lyon, Lyon, France; McGill University, Canada

## Abstract

Pattern recognition receptors (PRR), like Toll-like receptors (TLR) and NOD-like receptors (NLR), are involved in the detection of microbial infections and tissue damage by cells of the innate immune system. Recently, we and others have demonstrated that TLR2 can additionally function as a costimulatory receptor on CD8 T cells. Here, we establish that the intracytosolic receptor NOD1 is expressed and functional in CD8 T cells. We show that C12-iEDAP, a synthetic ligand for NOD1, has a direct impact on both murine and human CD8 T cells, increasing proliferation and effector functions of cells activated via their T cell receptor (TCR). This effect is dependent on the adaptor molecule RIP2 and is associated with an increased activation of the NF-κB, JNK and p38 signaling pathways. Furthermore, we demonstrate that NOD1 stimulation can cooperate with TLR2 engagement on CD8 T cells to enhance TCR-mediated activation. Altogether our results indicate that NOD1 might function as an alternative costimulatory receptor in CD8 T cells. Our study provides new insights into the function of NLR in T cells and extends to NOD1 the recent concept that PRR stimulation can directly control T cell functions.

## Introduction

Pattern recognition receptors (PRR) are involved in the detection of microbial infections as well as tissue damage in mammals. They are expressed by a variety of cell types in which they sense danger signals through the recognition of pathogen-associated molecular patterns (PAMPs) or endogenous damage-associated molecular patterns (DAMPs) [1]. Among the different families of PRR, Toll-like receptors (TLR) are membrane receptors able to sense extracellular microbial components such as lipopeptides, lipopolysacharride or flagellin through TLR-2, -4 and -5 respectively, as well as endosomal nucleic acid motifs by TLR-3, -7/8 and -9 [2]. TLR engagement in myeloid and epithelial cells leads to pro-inflammatory cytokine production through the activation of NF-κB, MAPK and Interferon Regulatory Factor pathways via the adaptor molecule MyD88 for all TLR except TLR3, and via TRIF for TLR3 and partially for TLR4 [3]–[4]. NOD-like receptors (NLR) are another family of PRR localized in the cytosol. Among them, NOD1 and NOD2 sense specific bacterial molecules, γ-D-glutamyl-*meso*-diaminopilemic acids (iE-DAP) and muramyl dipeptide (MDP) respectively, which are produced during the synthesis and/or the degradation of bacteria cell wall peptidoglycan [5]–[7]. NOD1 or NOD2 stimulation induces secretion of pro-inflammatory cytokines through the activation of the NF-κB and MAPK signaling pathways via the adaptor molecule RIP2 [8]. In contrast, recognition of cytosolic danger signals by other NLR, such as IPAF, NAIP5 and NALPs, leads to the formation of multiprotein complexes called inflammasomes, responsible for the activation of caspase-1, a protease required for processing and activation of the pro-inflammatory cytokines IL-1β and IL-18 [9].

PRR are highly expressed by cells of the innate immune system and have been mostly studied in myeloid cells such as macrophages and dendritic cells (DC). PRR agonists promote DC maturation, enhancing antigen presentation, costimulatory molecule expression and pro-inflammatory cytokine production. Thus, PRR stimulation plays an important role in indirectly controlling T cell activation [10]. Additionally, numerous reports have established that T cells also express certain TLR [11]–[12] and that TLR ligands can directly enhance activated T cell clonal expansion, conventional T cell effector functions [13]–[28] and can positively or negatively modulate regulatory T cell suppressive functions depending on the TLR ligand [29]–[37]. In particular, we and others have shown that TLR2 is expressed by CD8 T cells and that TLR2 engagement directly enhances proliferation, survival, pro-inflammatory cytokine secretion and cytotoxic functions of CD8 T cells activated via their T cell receptor (TCR) [17], [27]. TLR2 engagement on differentiated cytotoxic T lymphocytes also augments their antitumor activity [22]. Furthermore, TLR2 engagement on CD8 T cells can replace costimulatory signals provided by mature dendritic cells to induce an optimal activation [17], and TLR2 stimulation decreases CD8 T cell activation threshold for the TCR signal strength, enabling the generation of functional memory cells in response to a low affinity antigen [25]. These studies indicate that TLR2 functions as an alternative costimulatory receptor on CD8 T cells. Finally, the physiological importance of the direct impact of TLR2 ligands on CD8 T cells has been established *in vivo* by the demonstration that TLR2 deficiency in CD8 T cells impairs clearance of the bacteria Listeria monocytogenes [28], and that TLR2 plays a critical T cell-intrinsic role in CD8 T cell expansion and memory formation during an infection by vaccinia virus [26]. Regarding NLR, accumulating evidence shows expression of some of these receptors by human and murine T cells [38]–[41]. Yet, NLR potential function in T lymphocytes remains unclear. The group of G. Nuñez reported an intrinsic role for NOD2 in murine CD4 T cells, showing *in vitro* and *in vivo* a defect in activated NOD2^−/−^ CD4 T cell proliferation and secretion of IL-2 and IFN-γ in comparison to WT CD4 T cells [42]. However, two independent laboratories did not reproduce these results [43]–[44]. Finally, two recent reports showed that direct stimulation with the NOD2 ligand MDP protects human FOXP3^+^ T cells from death receptor Fas-mediated apoptosis [45] and increases IFN-γ secretion by TCR-activated γδ T cell [46].

Here, we investigate the function of NLR in CD8 T cells. We demonstrate that the receptor NOD1 can function as an alternative costimulatory receptor in murine and human CD8 T cells, and that direct NOD1 and TLR2 stimulations can cooperate to enhance TCR-mediated activation.

## Results

### NOD1 is expressed by CD8 T cells

**Figure 1:**
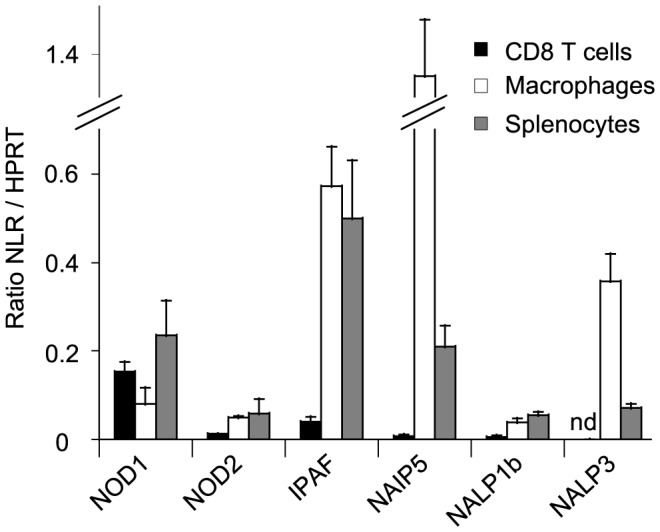
NOD1 is expressed by CD8 T cells. NLR mRNA expression assessed by quantitative RT-PCR in murine CD8 T cells (black bars), macrophages (white bars) and splenocytes (grey bars) (nd: not detected). Results are the mean expression of NLR relative to HPRT ± SD of 3 independent experiments.

To study the potential function of NLR within CD8 T cells, we analyzed mRNA expression of NOD1, NOD2, IPAF, NAIP5, NALP1b and NALP3 in murine CD8 T cells by quantitative RT-PCR. We clearly detected NOD1 mRNA expression in CD8 T cells (Figure 1) whereas NOD2, IPAF, NAIP5 and NALP1b mRNA levels were low and NALP3 mRNA was undetectable (Figure 1). NOD1 mRNA expression in CD8 T cells was comparable to NOD1 expression in both splenocytes and macrophages (Figure 1) in which NOD1 function has been described [47]. Thus, consistently with previous reports [38]–[41], our results show that NOD1 mRNA is expressed by murine CD8 T cells.

### NOD1 ligand directly increases TCR-mediated proliferation and effector functions

**Figure 2:**
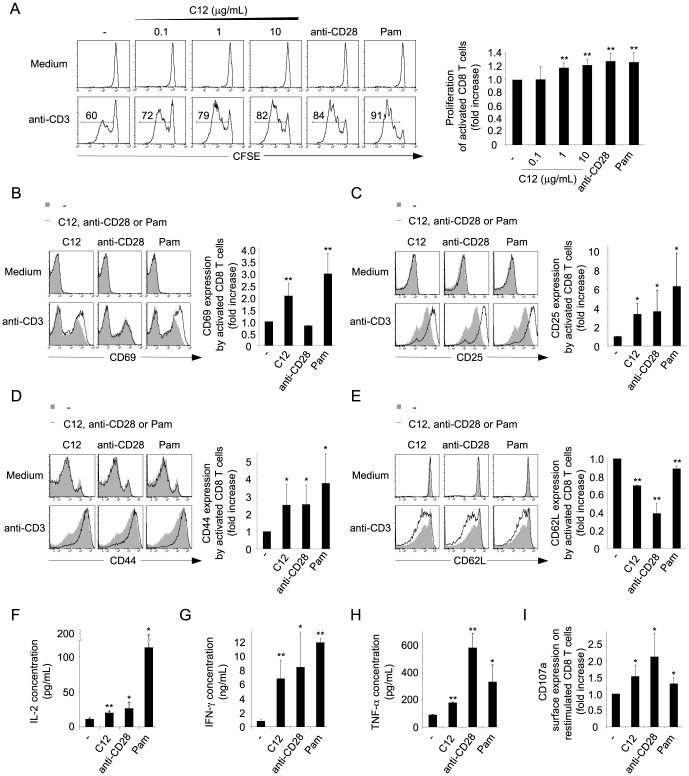
NOD1 ligand directly increases TCR-activated CD8 T cell proliferation and effector functions. (A) Flow cytometry assessment of the proliferation of CFSE stained murine CD8 T cells cultured for 72 h with or without anti-CD3 antibody, in the absence or presence of a dose range of C12, anti-CD28 or TLR2 ligand Pam. The percentage of proliferating cells is indicated within the histograms. The column graph represents the mean fold increase of anti-CD3 stimulated CD8 T cell proliferation in the different conditions, in comparison to the control condition anti-CD3 alone, ± SD from 5 independent experiments. (B–E) Flow cytometry assessment of CD69 (B) expression by CD8 T cell after 20 h of culture and of CD25 (C), CD44 (D) and CD62L (E) expression after 48 h of culture in medium containing or not anti-CD3 antibody, in the absence (solid grey) or presence of C12, anti-CD28 or Pam (black lines). The column graphs represent the mean fold increase of anti-CD3 stimulated CD8 T cell expression level of the different activation markers in the different conditions, in comparison to the control condition anti-CD3 alone, ± SD from 3 independent experiments. (F–H) Determination of IL-2, IFN-γ and TNF-α concentrations in CD8 T cells supernatants following 48 h of activation with anti-CD3, in the absence or presence of C12, anti-CD28 or Pam. Results are the mean concentrations of cytokines determined ± SD from 3 independent experiments. (I) Flow cytometry assessment of the surface expression of CD107a by CD8 T cells activated for 72 h with anti-CD3 in the absence or presence of C12, anti-CD28 or Pam, and restimulated for 4 h with anti-CD3. The column graph represents the mean fold increase of CD8 T cell surface expression level of CD107a in the different conditions, in comparison to the control condition anti-CD3 alone, ± SD from 3 independent experiments. (* = p<0.05 and ** = p<0.01; Student *t* test).

We next assessed the consequences of NOD1 stimulation in both resting or TCR-activated CD8 T cells. To avoid any indirect impact of NOD1 ligand on CD8 T cells via contaminating cells like antigen presenting cells, we sorted murine CD8 T cells by flow cytometry. Highly pure CD8 T cells (≥99%) were subsequently cultured in the presence or absence of anti-CD3 antibody, in medium supplemented or not with a dose range of a synthetic NOD1 ligand, C12-iEDAP (C12). After 72 h of culture in absence of anti-CD3, no cell division was detected (Figure 2A). In contrast, 60% of cells activated with anti-CD3 alone had undergone cell division and the percentage of dividing CD8 T cells was significantly increased with the addition of C12, reaching more than 80% in presence of 10 µg/mL of NOD1 ligand (Figure 2A). This effect of C12 was associated with a significantly increased expression of the activation markers CD69, CD25 and CD44 by activated CD8 T cells (Figure 2B–2D) and with a significant down-regulation of CD62L (Figure 2E). Again, stimulation of NOD1 in resting cells had no impact on the expression of these molecules (Figure 2B–2E).

To further investigate the direct effect of NOD1 ligand in CD8 T cells, we assessed the impact of C12 on the secretion of IL-2, IFN-γ and TNF-α by TCR-activated cells. After 48 hours of culture, significantly more IL-2, IFN-γ and TNF-α were secreted by T cells activated in presence of anti-CD3 and C12 in comparison to cells activated in presence of anti-CD3 alone (Figure 2F–2H). In addition, we analyzed the cytotoxic capacities of CD8 T cells activated in presence or absence of NOD1 ligand by assessing the surface expression of CD107a reflecting exocytosis of lytic granules [48]–[49]. We observed that presence of NOD1 ligand during CD8 T cell activation increased the surface expression of the degranulation marker following 4 h of restimulation with anti-CD3 (Figure 2I).

Altogether, our results demonstrate that NOD1 is functionally expressed in murine CD8 T cells and that NOD1 stimulation enhances CD8 T cell activation, increasing cell proliferation as well as effector functions. The observed effect of C12 on CD8 T cells was similar to the costimulation induced either by anti-CD28 or by the TLR2 ligand Pam_3_CSK_4_ (Pam) (Figure 2A–2I). These data suggest that NOD1 might function as an additional alternative costimulatory receptor in CD8 T cells.

### C12 costimulatory effect is NOD1- and RIP2-dependent and is associated with activation of NF-κB, JNK and p38 signaling pathways

**Figure 3:**
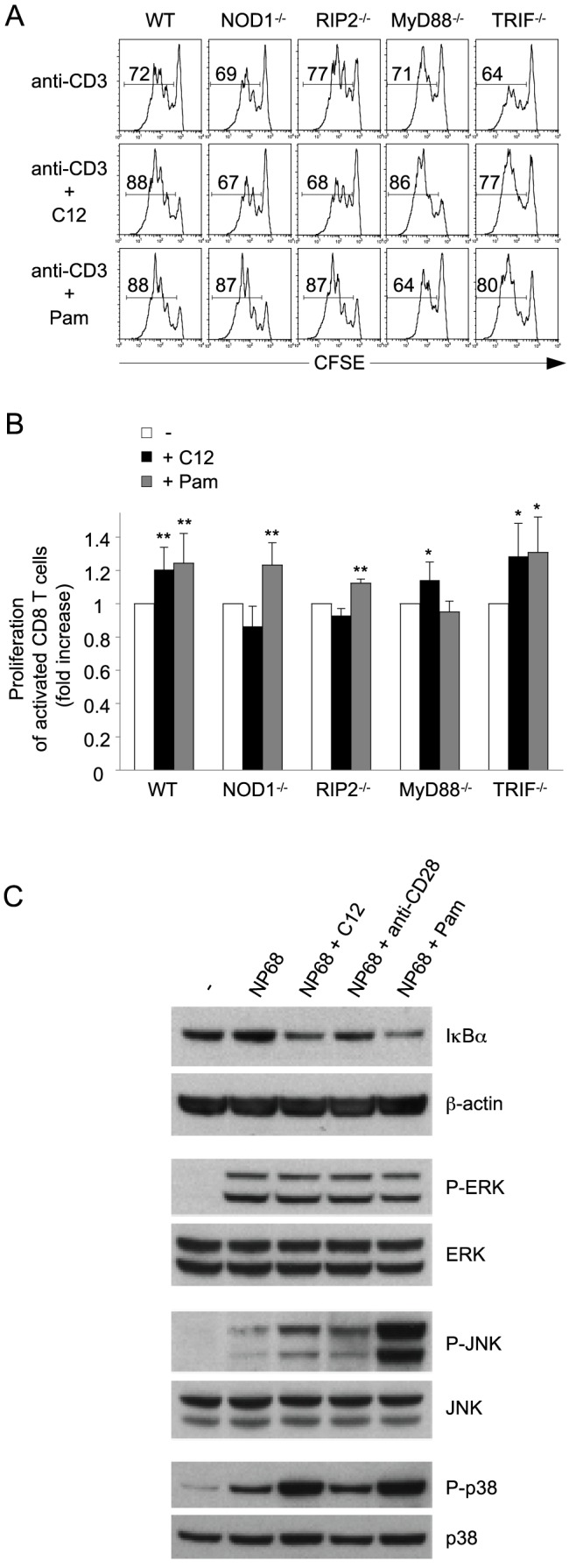
C12 effect on activated CD8 T cells is NOD1- and RIP2- dependent, and is associated with activation of the NF-κB, JNK and p38 signaling pathways. (A–B) Flow cytometry assessment of the proliferation of CFSE stained WT, NOD1^−/−^, RIP2^−/−^, MyD88^−/−^ and TRIF^−/−^ CD8 T cells activated for 72 h with anti-CD3, in the absence or presence of C12 or Pam. (A) The percentage of proliferating cells is indicated within the histograms. (B) The column graph represents the mean fold increase of anti-CD3 stimulated CD8 T cell proliferation in the different conditions, in comparison to the control condition anti-CD3 alone, ± SD from 3 independent experiments. (C) Determination by western blotting of IκBα, β-actin, Phospho-ERK (P-ERK), total ERK, Phospho-JNK (P-JNK), total JNK, Phospho-p38 (P-p38) and total p38 protein levels in F5 CD8 lymphoblasts cultured for 30 minutes in medium alone or with 1 nM of their specific antigenic peptide, NP68, in the absence or presence of C12, anti-CD28 or Pam. Results are representative of 3 independent experiments.

To characterize the molecular mechanisms involved in C12 mediated costimulation, WT, NOD1^−/−^, RIP2^−/−^, MyD88^−/−^ and TRIF^−/−^ CD8 T cells were sorted by flow cytometry and activated in the presence or absence of C12 or Pam (Figure 3A–3B). As expected, both ligands significantly increased WT CD8 T cell proliferation in response to CD3 stimulation and Pam costimulatory effect was abolished in MyD88^−/−^ CD8 T cells. C12 costimulatory effect was abolished in NOD1^−/−^ and RIP2^−/−^ CD8 T cells but not in MyD88^−/−^ or TRIF^−/−^ cells (Figure 3A–3B). These results show that C12 costimulatory effect in activated CD8 T cells is specifically mediated by NOD1 and RIP2 and not by TLR.

We next investigated the signaling pathways triggered by NOD1 stimulation in activated CD8 T cells. To address this question, we used CD8 T lymphoblasts transgenic for the TCR F5 that recognize specifically the antigenic peptide NP68 from A/NT/60/68 influenza virus as previously described [25]. Costimulation of activated CD8 T cells with C12 increased NF-κB inhibitor IκBα degradation and enhanced both JNK and p38 phosporylation levels (Figure 3C). In contrast, C12 costimulation had no impact on ERK phosphorylation (Figure 3C). Thus, C12 costimulatory effect in CD8 T cells is associated with the activation of NF-κB, JNK and p38 signaling pathways but not the ERK pathway. Interestingly, the signaling pathways triggered by NOD1 stimulation in activated CD8 T cells are similar to those induce by the TLR2 ligand Pam (Figure 3C) whereas they differ from CD28 mediated costimulation that has no impact on p38 phosphorylation and only mildly enhances IκBα degradation and JNK phosphorylation (Figure 3C).

### NOD1 cooperates with TLR2 in both murine and human CD8 T cells

**Figure 4:**
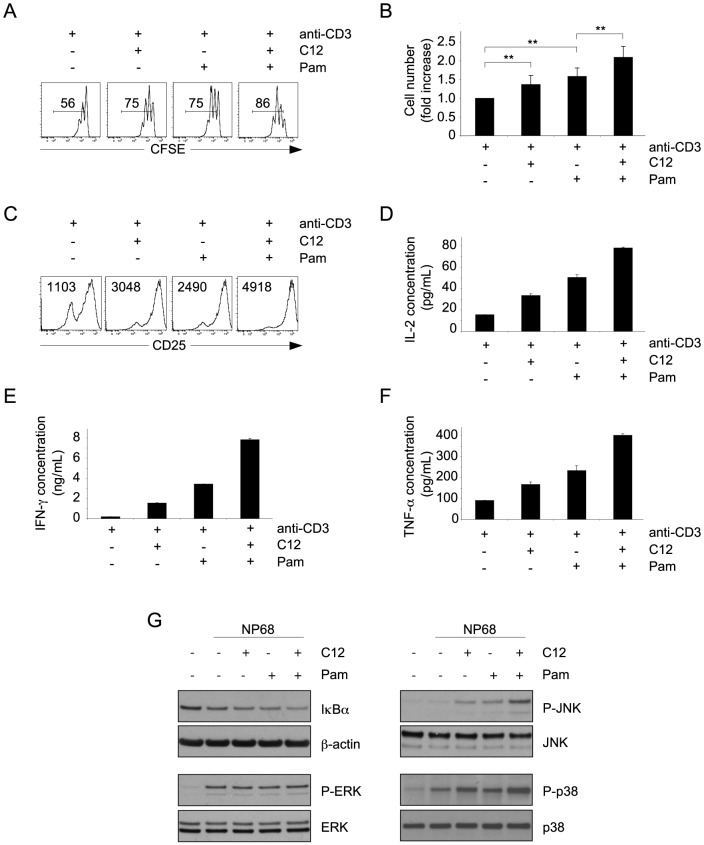
NOD1 cooperates with TLR2 to enhance TCR-mediated CD8 T cell activation. (A–C) Flow cytometry assessment of the proliferation (the percentage of proliferating cells is indicated within the histograms) (A), cell numbers (B) and CD25 expression (the mean fluorescence intensity of CD8 T cells is indicated within the histograms) (C) of CFSE stained murine CD8 T cells cultured for 48 h with anti-CD3, in the absence or presence of C12, Pam, or both C12 and Pam. (D–F) Determination of IL-2 (D), IFN-γ (E) and TNF-α (F) concentrations in the supernatants of CD8 T cells cultured for 48 h with anti-CD3, in the absence or presence of C12, Pam, or both C12 and Pam. (G) Determination by western blotting of IκBα, β-actin, Phospho-ERK (P-ERK), total ERK, Phospho-JNK (P-JNK), total JNK, Phospho-p38 (P-p38) and total p38 protein levels in F5 CD8 lymphoblasts cultured for 30 minutes in medium alone or with 1 nM of NP68, in the absence or presence of C12, Pam, or both C12 and Pam. (B) Cell number values are the mean fold increases of anti-CD3 stimulated CD8 T cell numbers in the different conditions, in comparison to the control condition anti-CD3 alone, ± SD from 4 independent experiments (** = p<0.01; Student *t* test). The other results are representative of 4 (A and C) or 3 (D, E, F and G) independent experiments.

Since NOD1-mediated costimulation of activated CD8 T cells was very similar to TLR2-mediated costimulation, we assessed the potential cooperation between the two PRR within CD8 T cells. After 48 h of activation with anti-CD3 alone, 50 to 60% of murine CD8 T cells had proliferated. The percentage of dividing cells increased up to 75% in the presence of C12 or Pam, while in the presence of both ligands, the percentage of proliferating cells was significantly increased to more than 85% (Figure 4A). This augmented proliferation was associated with a significant increase in cell number (Figure 4B), suggesting that the two PRR cooperate to enhance expansion as well as proliferation of activated CD8 T cells. The cooperation between NOD1 and TLR2 ligands also induced higher CD25 expression (Figure 4C) and improved CD8 T cell effector functions as reflected by enhanced secretion of IL-2, IFN-γ and TNF-α (Figure 4D–4F). Finally, this cooperation was associated with increased activation of the NF-κB, JNK and p38 signaling pathways (Figure 4G). Of note, we did not detect any cooperation between NOD1- and CD28-mediated costimulations (Figure S1).

**Figure 5:**
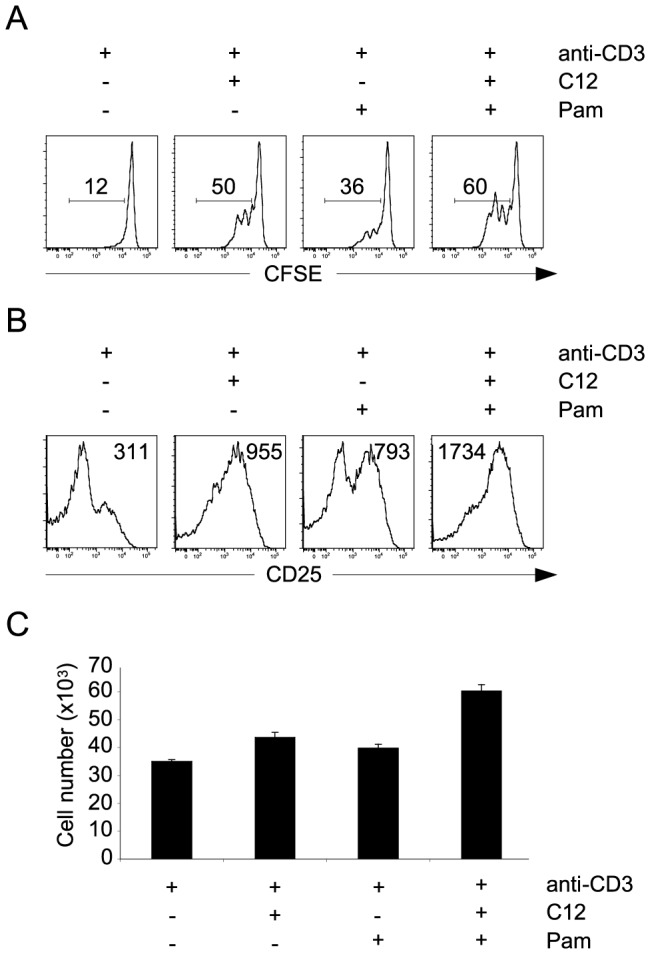
NOD1 cooperates with TLR2 to enhance TCR-mediated activation in human CD8 T cells. Flow cytometry assessment of the (A) proliferation (the percentage of proliferating cells is indicated), (B) CD25 expression (the mean fluorescence intensity of CD8 T cells is indicated) and (C) cell numbers of CFSE stained human CD8 T cells activated for 72 h with anti-CD3 in the absence or presence of C12, Pam, or both C12 and Pam. Results are representative of 3 independent experiments. CD8 T cell numbers are the mean cell number ± SD of triplicates.

Reinforcing our demonstration in murine CD8 T cells, we observed that NOD1 ligand also strongly increased proliferation and CD25 expression of CD3-stimulated human CD8 T cells (Figure 5A–5B). Furthermore, we showed that NOD1 and TLR2 ligands cooperate to enhance activated human CD8 T cell proliferation and expansion (Figure 5A and 5C).

Altogether our data suggest that detection of intracytosolic peptidoglycan by NOD1 and extracellular bacterial motifs and/or endogenous ligands by TLR2 are not redundant, but rather might increase CD8 T cells sensitivity to danger signals.

## Discussion

Our study establishes a previously undescribed role for the intracytosolic receptor NOD1 in both murine and human CD8 T cells. We demonstrate that NOD1 stimulation within CD8 T cells enhances their TCR-mediated activation, proliferation and effector functions. This effect on CD8 T cells is dependent on TCR engagement and is comparable to the costimulatory effects of CD28 [50] or TLR2 stimulation [17] previously described. These results suggest that NOD1 might function as an alternative costimulatory receptor in CD8 T cells. Our work contrasts with a recent study published by Asano J. and colleagues demonstrating a role for NOD1 in antigen cross-presentation to CD8 T cells [51]. This report showed that, independently of the presence or absence of the NOD1 ligand FK565, OT-1 CD8 T cells transferred into NOD1^−/−^ recipient mice displayed very little *in vivo* cytotoxic activity following immunization [51]. Hence, the authors inferred that NOD1 potential stimulation in CD8 T cell had no influence on cell activation. However, there was no evidence that the NOD1 ligand FK565 could reach the cytosol of CD8 T cells in this experimental setting. In addition, OT-1 cell basal antigenic stimulation was very poor [51] and our results demonstrate that TCR-mediated activation is required to reveal NOD1-mediated costimulation in CD8 T cells. Therefore the apparent controversy of our results can be explained by the differences between the two experimental systems.

Regarding the molecular mechanisms involved in NOD1-mediated costimulation, we demonstrate that C12 costimulatory effect in activated CD8 T cells is associated with enhanced activation of the NF-κB, p38 and JNK signaling pathways, which are involved in TCR-induced CD8 T cell survival, proliferation and differentiation [52]. In addition, we demonstrate that the effect of NOD1 ligand on CD8 T cells is dependent on the adaptor molecule RIP2, strongly supporting a function for NOD1 as a classical PRR in CD8 T cells. Moreover, our data provide new insights into the controversial function of RIP2 within T cells. Indeed, although the first studies of RIP2^−/−^ mice suggested that RIP2 might play a role in the TCR signaling pathway [53]–[55], recent reports showed no impact of RIP2 deficiency on CD4 or CD8 T cell activation via their TCR [56]–[58]. Consistent with the latter studies, we show that RIP2^−/−^ CD8 T cells present no defect in anti-CD3 induced proliferation in comparison to WT CD8 T cells (Figure 3A). Thus, our data confirm that RIP2 might not be involved in TCR signaling, but rather support a role for RIP2 as an essential mediator of NOD1-mediated costimulation in CD8 T cells.

Following the recent demonstrations that TLR engagement on T cells modulates their functions [12], our study extends to the intracytosolic NLR the capacity of PRR to directly affect proliferation and effector functions of activated CD8 T cells. Furthermore, as previously reported for cells of the innate immune system, we show that diverse PRR can be involved in sensing microbial danger signals in T cells. Hence, studying the potential functions of other PRR families like C-type lectins or CARD helicases in T cells would be very interesting.

Importantly, we also show that NOD1 can cooperate with TLR2 to strongly enhance proliferation, expansion and effector functions of activated CD8 T cells. These results extend for the first time to T cells the recent concept that different PRR families can collaborate to increase cellular responses to danger signal detection [59]. The contribution of these costimulatory effects to the efficiency of CD8 T cell-mediated immune responses against pathogens needs further investigation *in vivo*. Our study also provides new insights into the potential mechanisms for inflammatory and autoimmune diseases emergence. Indeed, CD8 T cells have been implicated in the initiation and/or development of various pathologies such as atopic dermatitis [60], asthma [61], rheumatoid arthritis [14] and inflammatory bowel disease [62]. NOD1 polymorphisms associated with increased activity of the receptor have been linked with augmented risks to develop atopic dermatitis, asthma and inflammatory bowel disease [63]–[67], and TLR2 has been involved in the development of allergic contact dermatitis as well as arthritis in different experimental models [68]–[70]. Our results suggest that in addition to the stimulation of the innate immune response, engagement of NOD1 in TCR-activated CD8 T cells, alone or in cooperation with TLR2 ligands, might exacerbate the T cell response and contribute to tolerance breaking. In this context, TLR2 might recognize extracellular bacterial lipopeptides or endogenous damage-associated molecules like products of the degradation of the extracellular matrix [71]–[72], heat shock proteins [73]–[74] or HMGB1 [75]. Regarding NOD1 ligands, recent studies have demonstrated translocation of peptidoglycan [76] as well as bacterial outer membrane vesicles [77] - able to deliver NOD1 ligand to the cytosol of non-phagocytic cells - from the gut bacteria to the organism. In particular, the group of J. Weiser revealed that translocation of NOD1 ligands from the gut to the blood and the bone marrow of mice occurred in absence of infection, establishing a mechanism for systemic modulation of immune responses by the microbiota [76]. These reports provide a mechanism for NOD1 stimulation in CD8 T cells in absence of T cell infection. Yet, the physiological importance of NOD1- and/or TLR2-mediated CD8 T cells costimulation in the development of autoimmune or inflammatory diseases needs to be evaluated *in vivo* using experimental models of such pathologies.

To conclude, this study reveals a previously undescribed role for NOD1 as a classical PRR in murine and human CD8 T cells. In addition to the recent demonstration that TLR can directly control T cell functions [12] and that MDP can be sensed by γδ T cells [46] and regulatory T cells [45], our results contribute to strongly challenge the dogma that restrict the role of germ-line encoded pattern recognition molecules to cells of the innate immune system.

## Materials and Methods

### Mice

C57BL/6 mice were purchased from Charles Rivers Laboratory. NOD1^−/−^, RIP2^−/−^, MyD88^−/−^ and F5 TCR transgenic mice were all from the C57BL/6 background. TRIF^−/−^ mice were of a mixed background. F5 TCR transgenic mice that express the TCR F5 recognizing specifically an H2-D^b^-restricted A/NT/60/68 influenza virus NP epitope (NP68) were described previously [25]. All mice were bred at the Plateau de Biologie Experimentale de la Souris (Experimental Murine Biology Platform) in specific pathogen-free conditions. Experimental procedures were submitted for approval to an institutional review board, the Regional Animal Experimentation Ethics Committee.

### Cell preparation and culture

Murine CD8 T cells from spleen and lymph nodes were first purified by magnetic beads using negative selection, then stained with anti-CD8 and anti-CD11c monoclonal antibodies (BD Pharmingen) and sorted by flow cytometry (FACS) according to CD8^+^ (for RT-PCR analysis) or CD8^+^ CD11c^−^ (for *in vitro* activation) expression. Purity of CD8 T cells was always greater than 99% (CD3^+^ CD8^+^ cells: 99.3±0.14%, F4/80^+^ cells: 0.016±0.00%, CD11c^+^ cells: 0.01±0.02%). Murine macrophages were collected from the peritoneal cavity of C57BL/6 mice through two washes with 3 mL of PBS. The collected cells were then sorted by flow cytometry according to F4/80 expression. Purity of macrophages was greater than 99%. Human CD8 T cells were purified from healthy donor peripheral blood lymphocytes by depletion of CD56^+^ cells using human CD56 microbeads (Miltenyi Biotec) followed by positive selection using human CD8 microbeads (Miltenyi Biotec). Purity of CD8 T cells was always greater than 89% (CD3^+^ CD8^+^ cells: 92.3±3.05%, CD1a^+^ cells: 0.015±0.005%, CD14^+^ cells: 0.06±0.02%). F5 lymphoblasts were obtained as previously described [25].

Murine CD8^+^ CD11c^−^ cells were cultured at 1×10^6^ cells/mL in complete RPMI 1640 medium with or without 1 µg/mL coated anti-CD3 antibody (2C11 clone), with or without C12-iEDAP (C12) (Invivogen) (10 µg/mL when not otherwise indicated), 2 µg/mL Pam_3_CSK_4_ (Pam) (EMC microcollections) or 2 µg/mL anti-CD28 (BD Pharmingen). Human CD8 T cells were cultured at 0.25×10^6^ cells/mL in RPMI 1640 medium 10% AB serum with 2.5 µg/mL coated anti-CD3 (UCHT-1 clone), with or without 10 µg/mL C12 or 2 µg/mL Pam.

### Proliferation, cell number, cell surface markers and cytokine detection by flow cytometry

Before culture, CD8 T cells were stained with CarboxyFluorescein Succinimidyl Ester (CFSE) (Molecular Probes). After 48 h of culture, cell numbers were measured by flow cytometry using Calibrite beads (BD Pharmingen) as standards as previously described [17]. After 48 h or 72 h of culture, cells were stained with anti-mouse or anti-human CD8, CD69, CD25, CD44 and CD62L monoclonal antibodies (BD Pharmingen). To assess the cytotoxic capacities of activated CD8 T cells, cells were collected after 72 h of culture, washed in complete RPMI and transferred into a new well coated with 1 µg/mL of anti-CD3 antibody. Cells were then restimulated for 4 h at 37°C in presence of soluble PE-conjugated anti-CD107a antibody (BD Pharmingen). Finally, CD107a surface expression by restimulated CD8 T cells was assessed by flow cytometry.

Fold increase in cell number, proliferation (% of dividing cells), or surface marker expression level (Mean fluorescence Intensity) were calculated as followed ; ex: Proliferation of activated CD8 T cells in presence of C12 (fold increase) = % of dividing CD8 T cells in presence of anti-CD3+C12/% of dividing CD8 T cells in presence of anti-CD3 alone.

Murine IL-2 and TNF-α concentrations in cell supernatants were determined using “mouse cytokine CBA” kits (BD Pharmingen) as previously described [78]. IFN-γ concentration in cell supernatants was determined using either “mouse cytokine CBA” kits (BD Pharmingen) or ELISA (R&D Systems).

### Real-time PCR and western blotting analysis

NLR mRNA expression was assessed in splenocytes and FACS-sorted macrophages and CD8 T cells by quantitative RT-PCR as previously described [17], using murine NOD1, NOD2, IPAF, NAIP5, NALP1b, NALP3 and HPRT specific primers (Table 1). NLR mRNA expression was normalized to the level of expression of the house-keeping gene HPRT. Analysis of the NF-κB, ERK, JNK and p38 signaling pathways was performed on F5 lymphoblasts cultured for 30 minutes in DMEM 6% FCS, with or without 1 nM of their specific antigenic peptide NP68 as previously described [25], with or without 10 µg/mL C12-iEDAP, with or without 10 µg/mL Pam_3_CSK_4_, using IκBα, phospho-ERK, ERK, phospho-JNK, JNK, phospho-p38 and p38 monoclonal antibodies (Cell Signaling Technology).

**Table 1:** Gene-specific primer sequences for real-time PCR.

Gene	Forward (5′ to 3′)	Reverse (5′ to 3′)	Amplicon size (bp)
NOD1	TAGACGGAAGCTGAAGGAACGC	ACCATCGGCTCTGCTCAAGTTC	120
NOD2	GGCTTCTCTGAAGAGGGCATCC	AGACAGGGAGGTGGCACAAAC	127
IPAF	GACTGCGGAGGTGGGAGATATG	GCACCTGGACTCCTGGATTTGG	112
**NAIP5**	CTTTCAAATTGTGAGTCTCTCATGGC	AAGGTCATGGCTTCAAAGCATCG	95
NALP1b	AAACGCCAGATAGGGTGAAGCC	GCCGGGCAGCAGGGATTATTC	96
NALP3	CTGGTCTGCTGGATTGTGTGC	GCCGTAGTGGTCTTGGAGGTC	86
HPRT	TCATTATGCCGAGGATTTGGA	CAGAGGGCCACAATGTGATG	119

### Statistical analysis

A two-tailed unpaired *t*-test was used to analyze the significance of the differences between the experimental conditions as indicated in the figure legends.

## Supporting Information

Figure S1 **NOD1 stimulation does not cooperate with CD28 engagement to enhance TCR-mediated CD8 T cell activation.** (A–C) Flow cytometry assessment of the proliferation (the percentage of proliferating cells is indicated within the histograms) (A), cell numbers (B) and CD25 expression (the mean fluorescence intensity of CD8 T cells is indicated within the histograms) (C) of CFSE stained murine CD8 T cells cultured for 48 h with anti-CD3, in the absence or presence of C12, anti-CD28, or both C12 and anti-CD28. (D–F) Determination of IL-2 (D), IFN-γ (E) and TNF-α (F) concentrations in the supernatants of CD8 T cells cultured for 48 h with anti-CD3, in the absence or presence of C12, anti-CD28, or both C12 and anti-CD28. Results are representative of 3 independent experiments.
(EPS)
